# High Fat Diet-Induced Skeletal Muscle Wasting Is Decreased by Mesenchymal Stem Cells Administration: Implications on Oxidative Stress, Ubiquitin Proteasome Pathway Activation, and Myonuclear Apoptosis

**DOI:** 10.1155/2016/9047821

**Published:** 2016-08-08

**Authors:** Johanna Abrigo, Juan Carlos Rivera, Javier Aravena, Daniel Cabrera, Felipe Simon, Fernando Ezquer, Marcelo Ezquer, Claudio Cabello-Verrugio

**Affiliations:** ^1^Laboratorio de Biologia y Fisiopatologia Molecular, Departamento de Ciencias Biologicas, Facultad de Ciencias Biologicas y Facultad de Medicina, Universidad Andres Bello, 8370146 Santiago, Chile; ^2^Millennium Institute on Immunology and Immunotherapy, 8370146 Santiago, Chile; ^3^Departamento de Ciencias Quimicas y Biologicas, Facultad de Salud, Universidad Bernardo O Higgins, 8370993 Santiago, Chile; ^4^Departamento de Gastroenterologıa, Facultad de Medicina, Pontificia Universidad Católica de Chile, 8330024 Santiago, Chile; ^5^Laboratorio de Fisiologia Integrativa, Departamento de Ciencias Biologicas, Facultad de Ciencias Biologicas y Facultad de Medicina, Universidad Andres Bello, 8370146 Santiago, Chile; ^6^Centro de Medicina Regenerativa, Facultad de Medicina, Clinica Alemana, Universidad del Desarrollo, 7710162 Santiago, Chile

## Abstract

Obesity can lead to skeletal muscle atrophy, a pathological condition characterized by the loss of strength and muscle mass. A feature of muscle atrophy is a decrease of myofibrillar proteins as a result of ubiquitin proteasome pathway overactivation, as evidenced by increased expression of the muscle-specific ubiquitin ligases atrogin-1 and MuRF-1. Additionally, other mechanisms are related to muscle wasting, including oxidative stress, myonuclear apoptosis, and autophagy. Stem cells are an emerging therapy in the treatment of chronic diseases such as high fat diet-induced obesity. Mesenchymal stem cells (MSCs) are a population of self-renewable and undifferentiated cells present in the bone marrow and other mesenchymal tissues of adult individuals. The present study is the first to analyze the effects of systemic MSC administration on high fat diet-induced skeletal muscle atrophy in the tibialis anterior of mice. Treatment with MSCs reduced losses of muscle strength and mass, decreases of fiber diameter and myosin heavy chain protein levels, and fiber type transitions. Underlying these antiatrophic effects, MSC administration also decreased ubiquitin proteasome pathway activation, oxidative stress, and myonuclear apoptosis. These results are the first to indicate that systemically administered MSCs could prevent muscle wasting associated with high fat diet-induced obesity and diabetes.

## 1. Introduction

Skeletal muscle is the most abundant tissue in the human body and has a wide variety of physiological functions. Therefore, muscle loss results not only in physical dysfunction but also in metabolic impairment [1, 2]. Related to this, recent studies have shown that obesity is associated with skeletal muscle loss and dysfunction and the development of muscle atrophy [3]. Indeed, mice fed with a high fat diet (HFD) develop the typical features of muscle wasting, such as weakness, the loss of muscle mass, and decreased fiber diameter [4].

Skeletal muscle is composed of a variety of fast and slow fiber types and subtypes (i.e., slow: I; fast: IIb, IId, and IIa; or mixtures) [5]. Moreover, muscle fibers are versatile and capable of changing phenotypic properties in response to muscle wasting, showing modifications in the expression of MHC isoforms. Therefore, when fast muscles atrophy, a fast-to-slow transition occurs (e.g., IIb→IId→IIa→I) [5, 6].

Ubiquitin proteasome pathway (UPP) overactivation, oxidative stress, and myonuclear apoptosis are some of the mechanisms involved in muscle atrophy induced by obesity and other causes [4, 7]. Overactivation of the UPP in muscle atrophy is characterized by an increased expression of two muscle-specific ubiquitin E3-ligase F-box proteins, muscle atrophy F-box (MAFbx)/atrogin-1 and muscle ring-finger protein 1 (MuRF-1). These proteins increase the ubiquitination of targets such as the myosin heavy chain (MHC), which are further degraded by the proteasome [8–11].

Oxidative stress is produced by increased reactive oxygen species (ROS) and/or decreased antioxidant mechanisms [12]. Oxidative stress has been associated with several models of muscle atrophy, including that induced by obesity [3, 13, 14]. Another mechanism involved in muscle atrophy is myonuclear apoptosis, which is related to muscle wasting induced by cachexia and obesity [4]. In atrophic muscle, several parameters of myonuclear apoptosis increase, such as the Bax/Bcl2 ratio, caspase-3 levels and activity, and apoptotic nuclei [15].

Stem cell-based interventions act through multiple mechanisms and provide a clear advantage when treating diseases with a complex pathophysiology, such as HFD-induced obesity. This type of intervention is arguably better in therapeutic terms than single-agent, drug-based treatments [16, 17]. Multipotent mesenchymal stromal cells, also referred to as mesenchymal stem cells (MSCs), are a heterogeneous adult stem cell population. These are a potentially ideal tool for stem cell-based interventions since they can be isolated from bone marrow and other mesenchymal tissues, including adipose tissue, dental pulp, the placenta, and the umbilical cord, and subsequently rapidly expanded* ex vivo* [18, 19]. As immunomodulatory cells, MSCs can limit inflammation in damaged tissue [20], produce a broad range of trophic factors that protect parenchymal cells from apoptotic death, and promote the proliferation and differentiation of endogenous precursors [21].

The present study is the first to provide evidence that systemic MSC administration can prevent the loss of muscle strength and mass, the decrease in fiber diameter and MHC protein levels, and the transition of fiber type in a murine model of HFD-induced muscle wasting. Prevention of the several parameters of muscle atrophy, among them UPP overactivation, increased ROS levels, and activation of myonuclear apoptosis, was also found in this model, which would explain the observed antiatrophic effects of MSC administration.

## 2. Materials and Methods

### 2.1. Animals

Male 12-week-old C57BL/10 mice were housed at a constant temperature (22 ± 2°C), with 60% relative humidity, and with a 12 : 12 light : dark cycle. Mice had* ad libitum* access to food and autoclaved water. Mice were fed with either a standard diet (control group, 10 cal% fat, 20 cal% proteins, and 70 cal% carbohydrates, Champion S.A., Chile) or a high fat diet (HFD group, 60 cal% fat, 20 cal% proteins, and 20 cal% carbohydrates, D12492 Research Diets Inc., USA). After 30 weeks of HFD feeding, mice were separated into two groups matched by average body weight. Then, for an additional eight weeks, one group received a vehicle treatment (HFD), while the other was treated with MSCs (HFD + MSCs). At the end of the 38-week experimental period, the mice were euthanized under anesthesia, and the tibialis anterior (TA) muscles were dissected, removed, and rapidly frozen and stored at −80°C until processing. All protocols were conducted in strict accordance with and with the formal approval of the Animal Ethics Committees of Universidad Andrés Bello and Universidad del Desarrollo.

### 2.2. Isolation and Administration of MSCs

Six-to-eight-week-old C57BL/10 male mice were sacrificed by cervical dislocation. Bone marrow cells were obtained by flushing the femurs and tibias with sterile PBS. After centrifugation, the cells were resuspended in an *α*-Minimum Essential Medium (Gibco, USA) supplemented with 10% selected fetal bovine serum (Hyclone, USA) and 80 *μ*g/mL gentamicin (Laboratorio Sanderson, Chile) and plated at a density of 1 × 10^6^ nucleated cells per square centimeter. Nonadherent cells were removed after 72 h by changing the medium. When the foci reached confluence, adherent cells were detached with 0.25% trypsin and 2.65 mM EDTA, centrifuged, and subcultured at 7,000 cells per square centimeter. After two subcultures, adherent cells were phenotypified and characterized according to the adipogenic and osteogenic differentiation potential, as previously described [6]. Then, 0.5 × 10^6^ MSCs were resuspended in 0.2 mL of 5% mouse plasma (vehicle) and administered via the tail-vein to anesthetized mice. Untreated mice were given 0.2 mL of vehicle.

### 2.3. Weightlifting Strength Test

At the end of the treatment, the muscle strength of the mice was measured through a weightlifting test, as previously described [22]. Briefly, the apparatus consisted in a series of increasingly long chain links attached to a ball of tangled fine wire. The number of links ranged from two to seven, with total weights between 15.5 and 54.1 g. Before performing the test and prior to treatments, the mice were trained once per day for two weeks. To perform the test, the mouse grasped the different weights with its forepaws and a score was assigned. The final score was calculated as the summation of the product between the link weight and the time the weight was held. The average of three measures from each mouse was normalized against body weight [23].

### 2.4. Contractile Properties

The TA muscles removed from sacrificed mice were placed in a dish containing oxygenated Krebs-Ringer solution (in mmol/L: NaCl: 118; NaHCO_3_: 25; D-glucose: 20; KCl: 4.7; CaCl_2_: 3.2; KH_2_PO_4_: 1.2; MgSO_4_: 1.2). The TA muscles were firmly tied with surgical silk at the bottom end of the tendon and at a portion of the knee bone. The muscle was transferred to a custom-built Plexiglas bath filled with oxygenated Krebs-Ringer solution that was thermostatically maintained at room temperature for optimal oxygen diffusion. The muscles were vertically aligned and tied directly between a fixed hook and a force transducer (MLT 1030/D, AD Instruments, USA). Two platinum plate electrodes were positioned in the organ bath so as to flank the length of the muscles. Muscles were field-stimulated by supramaximal square wave pulses (S48 Stimulator, Grass, USA), which were amplified to increase and sustain current intensity at a level sufficient for producing a maximum isometric tetanic contraction. Optimum muscle length (Lo) and stimulation voltage were determined from the micromanipulation of muscle length to produce maximum isometric twitch force. All stimulation parameters and contractile responses were controlled and measured using Power Lab 4/35 (AD Instruments, USA). All obtained data were computed with the LabChart analysis software (AD Instruments, USA). Maximum isometric tetanic force (Po) was determined from the plateau of the frequency-force relationship after successive stimulations between 10 and 150 Hz for 850 ms, with 2 min rest periods between the stimuli. After testing, the muscles were removed from the bath and the tendons and any adherent nonmuscle tissues were trimmed, blotted once on filter paper, and weighed. Muscle mass and Lo were used to calculate specific net force or the normalized force of each total muscle fiber cross-sectional area (mN/mm^2^) [24–27].

### 2.5. Blood Triglycerides, Cholesterol, Glucose, and Insulin Determination

After 4 h of fasting, mice were sacrificed and blood samples were collected. Serum triglycerides and cholesterol levels were determined in the ARCHITECT c8000 Clinical Chemistry Analyzer (Abbott, USA). Blood glucose levels were measured with the Accu-Chek Performance glucometer system (Roche Diagnostic, Germany). Plasma insulin levels were assayed using the Ultrasensitive Mouse-Insulin ELISA Kit (Mercodia, Sweden).

### 2.6. RNA Isolation, Reverse Transcription, and Quantitative Real-Time PCR

Total RNA was isolated from the TA muscles using TRIzol (Invitrogen, USA). The total RNA (1 *μ*g) was reverse transcribed to cDNA using random hexamers and Superscript II reverse transcriptase (Invitrogen, USA). TaqMan quantitative real-time PCR reactions were performed in triplicate, using an Eco Real-Time PCR System (Illumina, USA) with predesigned primer sets for mouse* atrogin-1*,* MuRF-1*, and the housekeeping gene* 18S* (TaqMan Assays-on-Demand, Applied Biosystems, USA). The mRNA expression was quantified using the comparative ΔCt method (2^−ΔΔCt^), with 18S as the reference gene. The mRNA levels were expressed relative to the mean expression in the vehicle-treated mice [23, 28].

### 2.7. Western Blot Analysis

The muscles were homogenized in Tris-EDTA buffer with a cocktail of protease inhibitors and 1 mM phenylmethanesulfonyl fluoride. Proteins were subjected to SDS-PAGE, transferred onto polyvinylidene difluoride membranes (Millipore, USA), and probed with mouse anti-MHC (1 : 1,000) (MF-20, Developmental Studies, Hybridoma Bank, University of Iowa, USA), mouse anti-tubulin (1 : 5,000), mouse anti-GAPDH (1 : 5,000), rabbit anti-Bax (1 : 500), rabbit anti-Bcl2 (1 : 500), rabbit anti-caspase-3 (1 : 500), rabbit anti-Ub proteins (1 : 500) (Santa Cruz Biotechnology, USA), rabbit anti-LC3B (1 : 1,500) (Cell Signaling, USA), and rabbit anti-p62 (1 : 1,500) (Abcam, USA). All immunoreactions were visualized by enhanced chemiluminescence (Thermo Scientific, USA). Images were acquired using Fotodyne FOTO/Analyst Luminary Workstation Systems (Fotodyne, Inc., USA).

### 2.8. Muscle Histology and Fiber Diameter Determination and Quantification

Fresh-frozen TA muscles were sectioned, and cryosections (8 *μ*m) were stained with hematoxylin and eosin (H&E) or Alexa-Fluor 594 tagged wheat germ agglutinin (WGA) (Life Technologies, USA) according to standard procedures. Wheat germ agglutinin-stained fibers were assessed through blind analysis using the Image J software (NIH, USA), with fiber sizes determined for seven randomly captured images from each experimental condition. Fibers were handled manually, and the minimal Feret diameter of each fiber was computed by the software [28, 29].

### 2.9. Immunohistochemical Analysis

For immunohistochemistry, fresh-frozen TA muscle cryosections (8 *μ*m) were fixed in acetone and incubated overnight in 1% bovine serum albumin in PBS with anti-MHC IIa (clone SC-71-s; 1 : 20) and anti-MHC IIb (clone BF-F3-s; 1 : 20) (Developmental Studies, Hybridoma Bank, University of Iowa, USA). Then, cryosections were blocked for 15 min in 3% methanol-H_2_O_2_ and incubated for 30 min with the Envision Dual Link System-HRP (Dako, USA). Enzyme activity was detected with a 3′,3′-diaminobenzidine tetrahydrochloride liquid system (Dako, USA). Nuclei were stained with hematoxylin [26, 30].

### 2.10. ROS Detection

For ROS detection, fresh-frozen TA muscle cryosections (8 *μ*m) were incubated for 30 min at 37°C with 10 *μ*M CM-H_2_DCF-DA dye (Molecular Probes, Eugene, Oregon, USA) in Hank's buffered salt solution. Then, the muscles were fixed in 4% paraformaldehyde in PBS for 10 min at room temperature. The nuclei were stained with Hoechst 33258 (1 : 5,000) in PBS. After rinsing, the muscles were mounted with a fluorescent mounting medium (Dako, USA) under a glass slide and viewed and photographed using the Motic BA310 epifluorescence microscope [31].

### 2.11. Terminal Deoxynucleotidyl Transferase Mediated dUTP Nick End Labelling (TUNEL) Technique

Breaks in the DNA strand as a result of endonuclease activity were detected by the TUNEL labelling technique using the commercial DeadEnd Colorimetric TUNEL Kit (Promega, USA). The label was developed with a DAB 3,3′-diaminobenzidine horseradish peroxidase substrate that produces a dark-brown reaction product. A blinded field quantification of TUNEL-positive nuclei was then performed on five randomly captured images of the TA taken from four mice and for each experimental condition [15].

### 2.12. Caspase-3 Activity

The activity of caspase-3 was determined in the TA protein extract using the commercial Caspase-3 Colorimetric Assay Kit (BioVision Inc., USA). A 405 nm absorbance value for each experimental condition was expressed as a fold of induction relative to control muscle [15].

### 2.13. Statistics

For statistical analysis, two-way analysis of variance (ANOVA) was used with a* post hoc* multiple-comparison Bonferroni test (Prisma). Differences were considered statistically significant at *P* < 0.05.

## 3. Results

### 3.1. Systemic Administration of Mesenchymal Stem Cells Reverts the Decreased Muscle Strength Induced by a High Fat Diet

**Figure 1:**
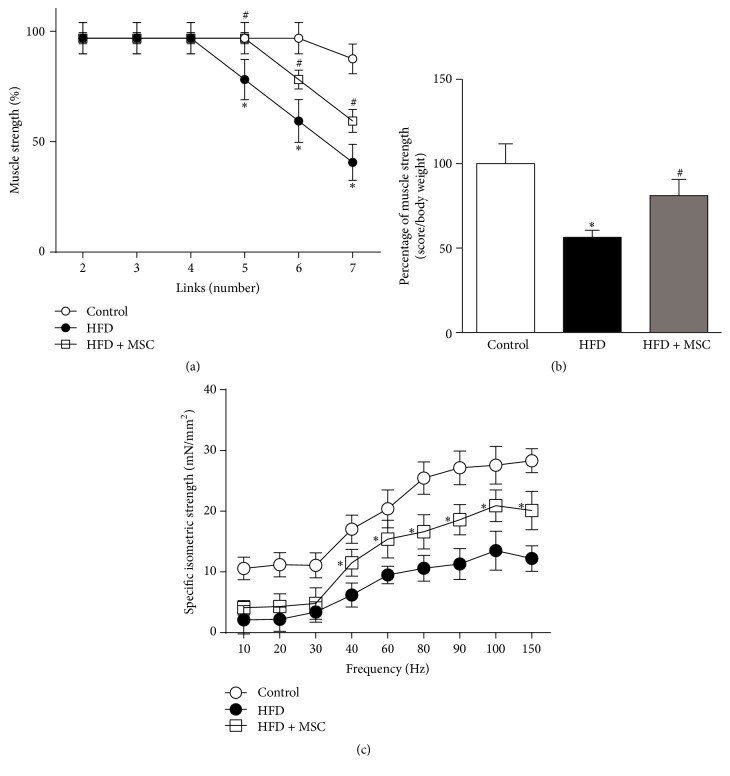
Mesenchymal stem cells (MSCs) administration inhibits the decreased muscle strength induced by a high fat diet (HFD) in mice. C57BL/10J male mice were fed with a standard chow (control) or HFD for 38 weeks. At week 30, a subgroup of HFD mice received MSC injected through the tail-vein. At week 38, all mice were subjected to the following: (a) A weightlifting test to determine limb muscle strength: values represent the percentage of muscle strength reached by the mice with each weight and correspond to the mean ± SD (*n* = 8; ^*∗*^ *P* < 0.05 versus control; ^#^ *P* < 0.05 versus HFD, two-way ANOVA). (b) A weightlifting test: the values represent the scores normalized by body weight. The values correspond to the mean ± SD (*n* = 8; ^*∗*^ *P* < 0.05 versus control; ^#^ *P* < 0.05 versus HFD, two-way ANOVA). (c) Maximal isometric strengths (mN/mm^2^) against stimulation frequencies (Hz) in the tibialis anterior (TA) muscles: values represent the mean ± SD (*n* = 8; ^*∗*^ *P* < 0.05 versus HFD, two-way ANOVA).

After receiving a HFD for 38 weeks, live mice evidenced decreased muscle strength, as evaluated by a weightlifting assay (Figure 1(a)). However, HFD + MSC mice showed a partial recovery of muscle strength after MSC treatment (Figures 1(a) and 1(b)).

Muscle strength was also measured through electrophysiological assays of tetanic force in isolated TA muscles. While the muscle force of HFD mice decreased in all of the assessed frequency ranges (Figure 1(c)), muscle strength was recovered, albeit not completely, following MSC treatment. This recovery reached approximately 50% for frequencies from 40 to 150 Hz and was without differences in lower frequencies.

These results suggest that MSC administration can prevent the decreased muscle strength induced by a HFD in mice.

### 3.2. Mesenchymal Stem Cells Prevent Fiber Type Transition, Decreased Fiber Diameter, and the Myosin Heavy Chain Levels Induced by a High Fat Diet

**Figure 2:**
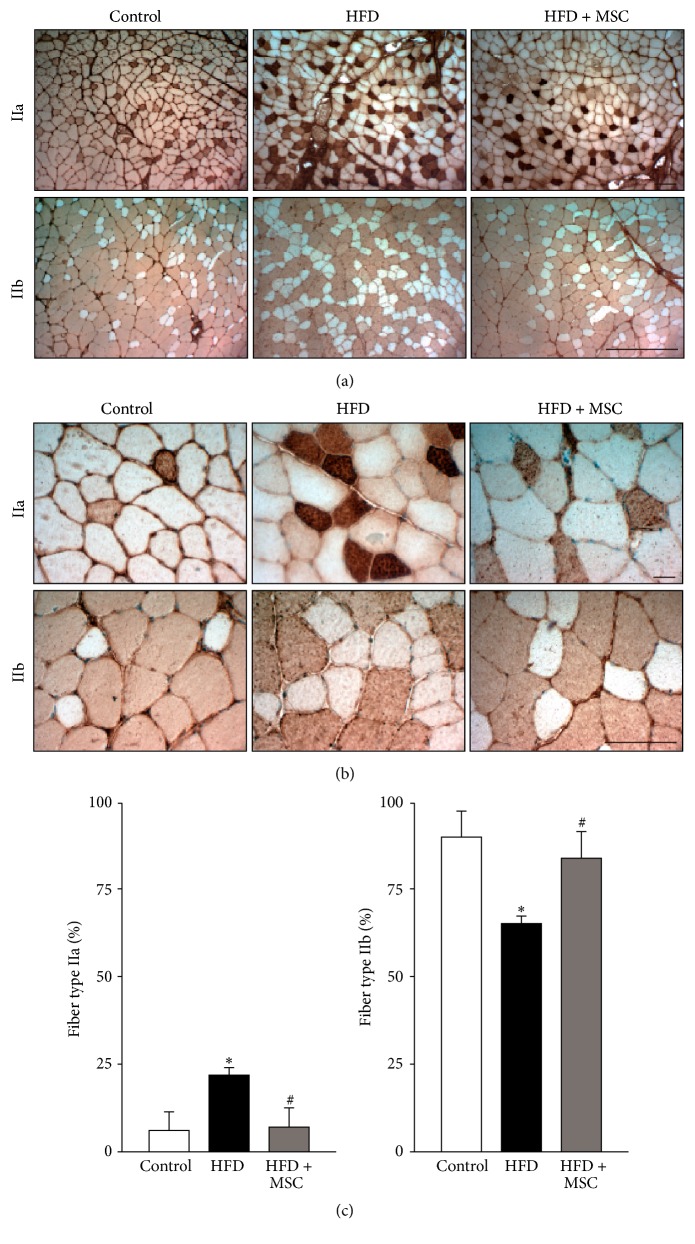
Systemic mesenchymal stem cells (MSCs) administration inhibits fiber type transitions in the tibialis anterior (TA) muscle of mice fed with a high fat diet (HFD). C57BL/10J male mice were fed with a standard chow (control) or HFD for 38 weeks. At week 30, a subgroup of HFD mice received MSC injected through the tail-vein. At week 38, all mice were sacrificed and the TA was analyzed to determine fiber type through the immunohistochemical detection of myosin heavy chain isoforms (IIa and IIb). Images obtained at 10x (a) and 40x (b) magnification show fiber types IIa (upper panel) and IIb (lower panel). Quantitative analysis of the fiber type is shown in (c). Graph representing the percentage of specific fiber types relative to the total fibers counted per field. Values represent the mean ± SD (*n* = 8; ^*∗*^ *P* < 0.05 versus control; ^#^ *P* < 0.05 versus HFD, two-way ANOVA).

The most abundant fiber type in the TA is IIb. When the TA is atrophied, IIb fibers transition towards IIa fibers [5, 6]. This was observed in the present study, where a HFD produced a shift to IIa fibers in affected mice, increasing the quantity of IIa fibers from 4.3 to 24.5% and decreasing the quantity of IIb fibers from 92.8 to 69.4% (Figures 2(a) and 2(b)). This transition was prevented in the HFD + MSC group, which showed a similar proportion of IIb fibers as the control group (Figures 2(a) and 2(b)). The graphs in Figure 2(c) show the quantitative analysis of Figures 2(a) and 2(b).

**Figure 3:**
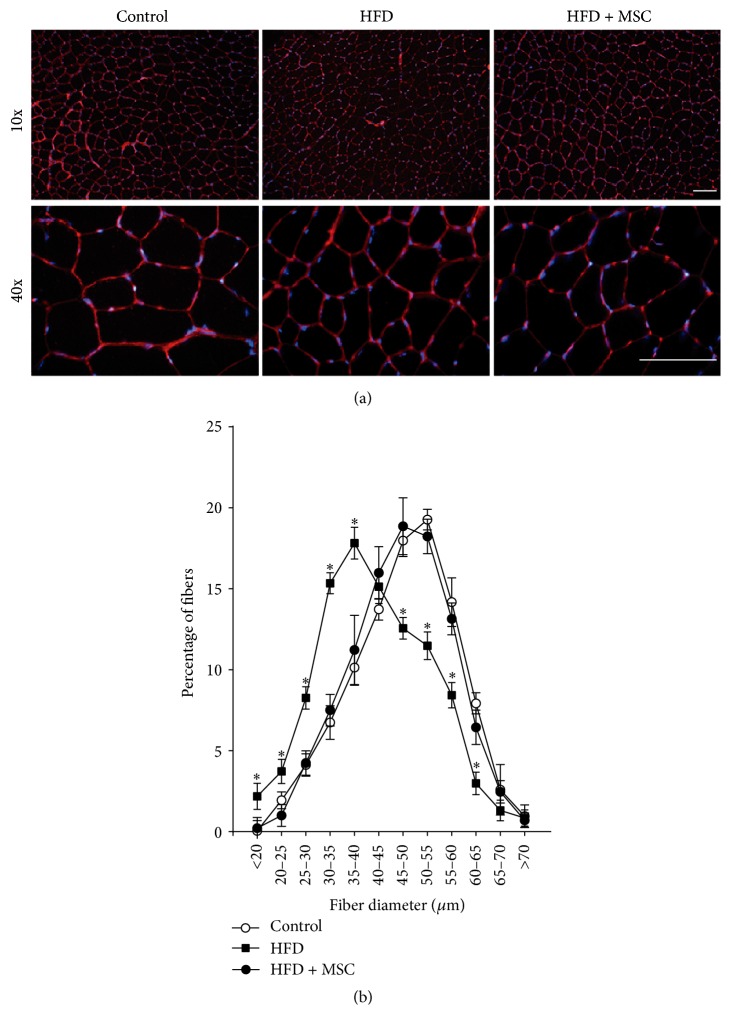
Mesenchymal stem cells (MSCs) administration prevents the decreased fiber diameter of the tibialis anterior (TA) muscle in high fat diet- (HFD-) induced skeletal muscle atrophy. TA muscles were obtained from C57BL/10J male mice fed with standard chow (control), a HFD, or a HFD-MSC treated. Muscle cross sections were stained with wheat germ agglutinin to delimit muscle fiber sarcolemma. (a) Images were obtained at 10x and 40x magnification. Bars correspond to 150 *μ*m. (b) Minimal Feret diameters were determined in TA cross sections from (b). Fiber diameters were grouped and ranged from 0 to 70 *μ*m. Values are expressed as a percentage of the total quantified fibers. Values correspond to the mean ± SD (*n* = 8; ^*∗*^ *P* < 0.05 versus control, two-way ANOVA).

The architecture of the TA muscles was conserved without evident changes and without foci of necrotic or damaged fibers (Supplemental Figure S1) (see Supplementary Material available online at http://dx.doi.org/10.1155/2016/9047821). However, when fiber size was evaluated (Figure 3(a)), the HFD mice showed a notable displacement towards smaller sized fibers, a situation that was reversed to the normal values in the HFD + MSC group (Figures 3(a) and 3(b)).

**Figure 4:**
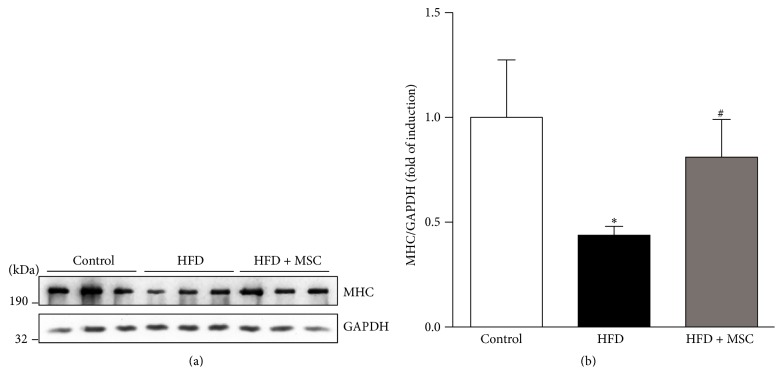
Systemic mesenchymal stem cells (MSCs) administration prevents the decreased myosin heavy chain (MHC) levels in the tibialis anterior (TA) muscle of mice fed with a high fat diet (HFD). C57BL/10J male mice were fed with a standard chow (control) or HFD for 38 weeks. At week 30, a subgroup of HFD mice received MSC injected through the tail-vein. After eight weeks, all mice were sacrificed and the TA was excised and homogenized to evaluate the following: (a) MHC protein levels through Western blot analysis (GAPDH levels were used as the loading control; molecular weight markers are shown in kDa) and (b) quantitative analysis of the experiments from (a). The levels of MHC normalized to GAPDH are expressed relative to control mice (^*∗*^ *P* < 0.05 versus control; ^#^ *P* < 0.05 versus HFD).

A characteristic feature of muscle atrophy is a decrease in the levels of the myofibrillar proteins such as MHC. In line with this, the HFD decreased MHC protein levels in the TA to only 40% of levels in control TA. In contrast, in HFD + MSC mice, the administration of MSC partially prevented lowered MHC levels, which reached 82% of the levels presented in control TA (Figures 4(a) and 4(b)).

### 3.3. Mesenchymal Stem Cell Administration Reverts Ubiquitin Proteasome Pathway Overactivation, the Increases in Reactive Oxygen Species, and the Myonuclear Apoptosis Induced by a High Fat Diet

**Figure 5:**
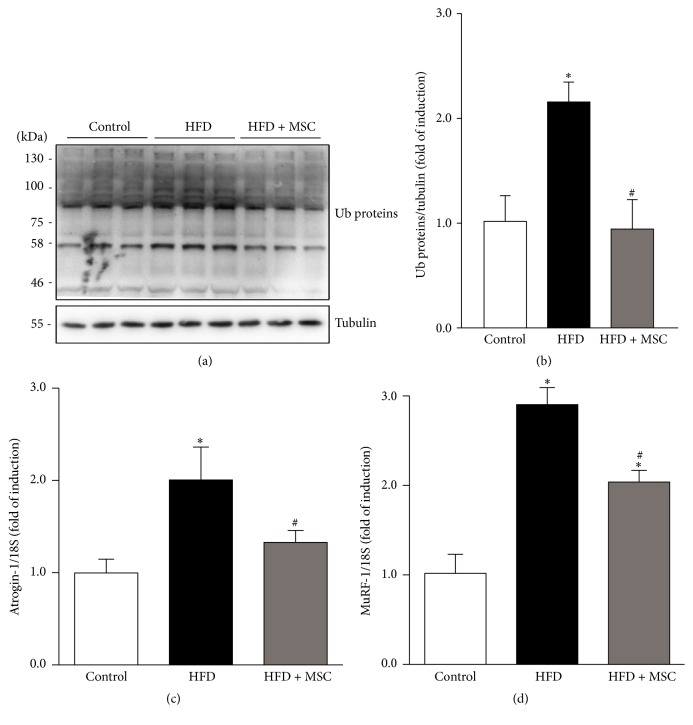
Mesenchymal stem cells (MSCs) administration inhibits increases in ubiquitinated proteins and atrogin-1 and MuRF-1 gene expression levels induced by a high fat diet (HFD) in mice. C57BL/10J male mice were fed with a standard chow (control) or HFD for 38 weeks. At week 30, a subgroup of HFD mice received MSC injected through the tail-vein. At week 38, all mice were sacrificed and the TA was excised and homogenized to evaluate the following: (a) total ubiquitinated (Ub) protein levels through Western blot analysis (tubulin levels were used as the loading control; molecular weight markers are shown in kDa) and (b) quantitative analysis of the experiments from (a). The levels of Ub normalized to tubulin are expressed relative to control mice (^*∗*^ *P* < 0.05 versus control; ^#^ *P* < 0.05 versus HFD). Detection of atrogin-1 (c) and MuRF-1 (d) mRNA levels through RT-qPCR using 18S as the reference gene. Expressions are shown as the fold of induction relative to the TA from control mice, and the values correspond to the mean ± SD (^*∗*^ *P* < 0.05 versus control; ^#^ *P* < 0.05 versus HFD).

Increased UPP activity was demonstrated by high levels of ubiquitinated proteins in the TA of HFD mice (Figures 5(a) and 5(b)). In contrast, HFD + MSC mice were able to decrease protein ubiquitination, reaching UPP activity levels similar to the control group. To corroborate this result,* atrogin-1* and* MuRF-1* expression, which increase under most muscle atrophy conditions, were also evaluated. A HFD induced increased* atrogin-1* and* MuRF-1* gene expression (Figures 5(c) and 5(d)). However, MSC treatment in HFD + MSC mice completely prevented increased* atrogin-1* expression and partially prevented increased* MuRF-1* expression in the TA (Figures 5(c) and 5(d)). When autophagy was evaluated, the levels and processing of LC3B and p62 did not show differences between the controls, HFD, and HFD + MSC mice (Supplemental Figures S2A, B, and C).

**Figure 6:**
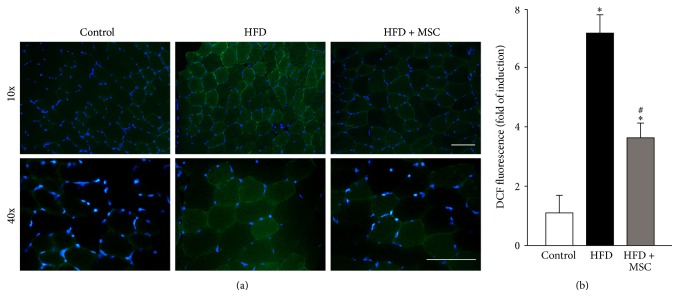
Systemic mesenchymal stem cells (MSCs) administration prevents increased reactive oxygen species (ROS) levels in the tibialis anterior (TA) of mice fed with a high fat diet (HFD). C57BL/10J male mice were fed with a standard chow (control) or HFD for 38 weeks. At week 30, a subgroup of HFD mice received MSC injected through the tail-vein. At week 38, all mice were sacrificed. (a) Cryosections obtained from the TA were incubated with a DCF probe for ROS detection through fluorescence microscopy. Nuclei were labelled via Hoechst staining. (b) Quantification of ROS levels from experiments showed in (a). The values are expressed as the fold of induction of the DCF probe intensity. Values correspond to the mean ± SD (*n* = 8; ^*∗*^ *P* < 0.05 versus control; ^#^ *P* < 0.05 versus HFD, two-way ANOVA).

The levels of ROS were also evaluated in the TA of HFD mice. For this, a DCF probe was used to detect (Figure 6(a)) and quantify (Figure 6(b)) ROS. HFD increased ROS levels 7.22-fold relative to the control, while MSC administration partially prevented this increase (3.83-fold relative to the control).

**Figure 7:**
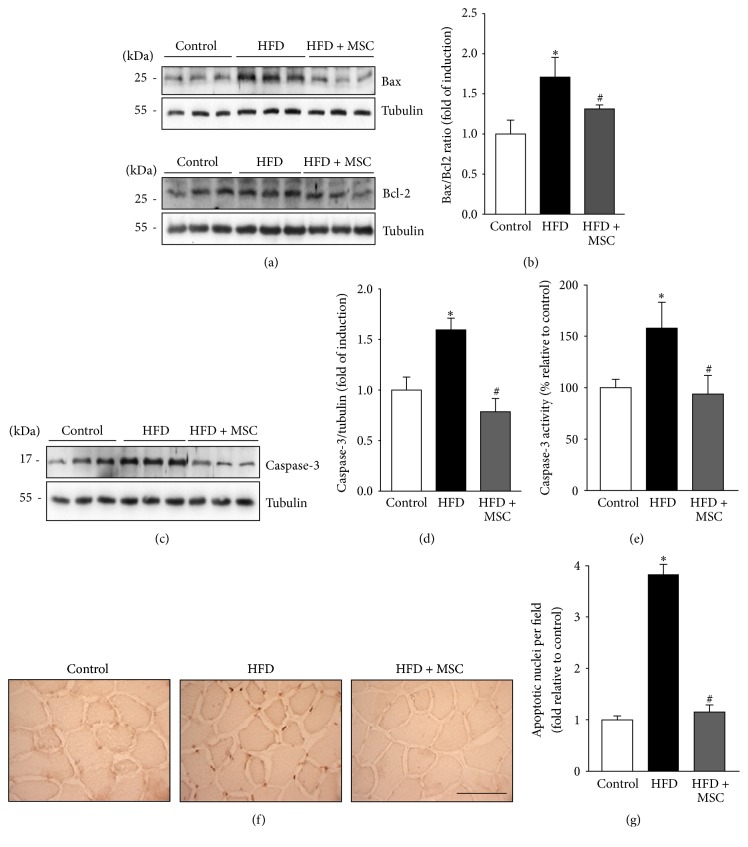
Mesenchymal stem cells (MSCs) administration inhibits the increased myonuclear apoptosis induced by a high fat diet (HFD) in mice. C57BL/10J male mice were fed with a standard chow (control) or HFD for 38 weeks. At week 30, a subgroup of HFD mice received MSC injected through the tail-vein. At week 38, all mice were sacrificed and the tibialis anterior (TA) was excised and homogenized to evaluate (a) Bax and Bcl2 protein levels through Western blot analysis; (b) Bax/Bcl2 ratio analysis (the values are expressed as fold of induction relative to the control and correspond to the mean ± SD (*n* = 8; ^*∗*^ *P* < 0.05 versus control; ^#^ *P* < 0.05 versus HFD, two-way ANOVA)); (c) cleaved caspase-3 levels detected by Western blot analysis; (d) quantification of caspase-3 levels from experiments shown in (c). The values are expressed as fold of induction relative to the control and correspond to the mean ± SD (*n* = 8; ^*∗*^ *P* < 0.05 versus control; ^#^ *P* < 0.05 versus HFD, two-way ANOVA). For (a) and (c), the levels of tubulin are shown as the loading control. The molecular weights are shown in kDa. (e) Caspase-3 activity was measured and expressed as fold of induction relative to the control, with values corresponding to the mean ± SD (*n* = 8; ^*∗*^ *P* < 0.05 versus control; ^#^ *P* < 0.05 versus HFD; two-way ANOVA). (f) Cryosections of TA were used to perform the TUNEL assay. The bar corresponds to 50 *μ*m. (g) Blinded quantification of TUNEL-positive nuclei per field in five randomly captured images. The values are expressed as the fold of induction relative to the control and correspond to the mean ± SD (*n* = 8; ^*∗*^ *P* < 0.05 versus control; ^#^ *P* < 0.05 versus HFD, two-way ANOVA).

Another mechanism recently described in relation to HFD-induced muscle wasting is myonuclear apoptosis [4]. Proapoptotic Bax levels and antiapoptotic Bcl2 levels were measured (Figure 7(a)). The Bax/Bcl2 ratio increased in the TA of HFD mice as compared to the control group (Figure 7(b)). However, HFD + MSC mice presented a decreased Bax/Bcl2 ratio in the TA as compared to the HFD group. Additionally, HFD-induced caspase-3 protein levels (Figure 7(c)) were decreased by MSC administration (Figure 7(d)). Similarly, MSC injection decreased HFD-induced caspase-3 activity in the TA (Figure 7(e)). Finally, apoptotic nuclei were evaluated by TUNEL analysis (Figure 7(f)). The results revealed that a HFD increased the number of apoptotic nuclei in the TA (165.4% relative to the control), while MSC injection fully prevented this increase in HFD + MSC mice (Figure 7(g)). Together, these results indicate that MSC administration decreases myonuclear apoptosis in the TA of HFD mice.

## 4. Discussion

It is well reported that HFD impairs muscle function and, specifically, produces muscle atrophy [32–34]. HFD induces skeletal muscle atrophy through the induction of several mechanisms, such as UPP overactivation, increased oxidative stress, and the generation of myonuclear apoptosis [4, 7]. In this context, the present study corroborated that HFD can produce phenotypical changes in skeletal muscle, many of which also occur in muscle atrophy caused by other stimuli, such as by immobilization, sepsis, or cachexia. These changes include decreased muscle strength and fiber size, fiber type transitions (e.g., IIb→IIa), the downregulation of MHC levels, the upregulation of atrogenes such as atrogin-1 and MuRF-1, and increased oxidative stress and myonuclear apoptosis. Interestingly, all of these features of HFD-induced muscle wasting were prevented when MSC was systemically administrated to mice.

One feature of obesity is an increase of some circulating factors that can affect muscle function and structure, producing muscle atrophy. These factors include angiotensin II and TNF-*α* [7, 35, 36]. Thus, the next step in our model of study is to know if the MSC administration alters the circulating and tissue levels of angiotensin II and TNF-*α*. The mechanisms through which angiotensin II and TNF-*α* induce muscle wasting include increased oxidative stress, UPP overactivation, and myonuclear apoptosis [7, 37–39]. These studies are in line with the present results, which show that MSC treatment prevented oxidative stress, UPP overactivation, and myonuclear apoptosis. Building on this result, it is important that future studies elucidate the possible participation of the angiotensin II and TNF-*α* induced pathways in the improvements observed when MSC is given to obese mice.

Despite the fact that one of the effects observed by MSC treatment is the decrease of myonuclear apoptosis, the cell type responsible for or sensible to this effect is not studied. We cannot rule out the possibility that apoptotic signaling may have occurred by a combination of muscle (fiber or satellite cells) and nonmuscle cells (e.g., endothelial cells and resident fibroblast) that reside inside muscles* in vivo*. Moreover, we cannot be sure that apoptotic myonuclei are intramuscular or associated with satellite cells. However, considering that the main and more abundant cell type in skeletal muscle is the muscle fibers and the evidences that describe the fibers as main source of apoptotic myonuclei in models of muscle atrophy by unloading [40], then we can speculate that at least this cell type significantly contributes to the changes in apoptotic signaling induced by high fat diet (HFD) and therefore it is sensitive to MSC treatment. This suggestion is supported by other studies that show the muscle fiber as the main cell source of the myonuclear apoptosis induced by HFD [4, 41]. Despite these evidences, we cannot discard that part of the atrophic effect observed under HFD can be attributed to another cell type, specifically to satellite cells whose dysfunction has been reported in atrophied muscles [42, 43], opening another cell target to the effect of MSC administration. Thus, further studies, such as specific immunolocalization of apoptotic marker, must be performed to detect and clarify the cell types responsible for the myonuclear apoptosis in the skeletal muscle under HFD, evaluating in addition the effect of MSC treatment.

The use of stem cell therapy to improve muscle disorders has been previously evaluated in models of muscle regeneration or dystrophy, mainly using the graft strategy [44, 45]. However, the efficiency of these procedures has been poor. In turn, the present results indicate that systemic MSC administration improves skeletal muscle atrophy associated with a HFD. Importantly, the effects of MSC administration were not related to obesity reversion, since HFD mice remained obese, hypercholesterolemic, hyperglycemic, and hyperinsulinemic (Supplemental Table S1).

Also worth noting, the MSCs incorporated into the muscle tissue of HFD mice were undetected in posttreatment analyses (data not shown). Diverse studies have shown that <1% of systemically administered MSCs remain present one week after administration in any organ, including the lungs, heart, kidneys, liver, spleen, and gut [16, 46, 47]. However, the clinical benefits associated with MSC administration can be observed for a much longer period. Considering this, it is possible to speculate that the impaired muscle function and features of muscle atrophy were prevented by some indirect event dependent on the MSCs. For example, MSCs secrete a broad range of bioactive growth factors, such as VEGF, bFGF, IGF, HGF, and EGF [21]. Therefore, MSCs could provide trophic support for injured tissue by modifying the microenvironment, thus inducing local precursor proliferation and differentiation to improve damaged tissue irrigation and prevent parenchymal cell apoptosis [17, 21]. Considering this, the impaired muscle function and features of muscle atrophy could be prevented by some indirect or paracrine event dependent on MSCs, as has been previously found in relation to the secretion of trophic factors [21, 48]. If this is the mechanism by which the effects of MSCs occur, it is possible that MSCs increase the secretion of trophic factors such as EGF and bFGF [21]. In skeletal muscle, EGF and bFGF also stimulate growth and prevent muscle atrophy [49, 50].

Through MSC administration, the muscle function affected by sepsis-induced skeletal muscle wasting was partially recovered, mainly through improving the function of satellite cells, the primary cell population responsible for repairing skeletal muscle [51]. Considering differences in MSC administration between Gao et al. [46] and the present study, it is possible that, in the present model of HFD-induced muscle wasting, MSC treatment could induce the secretion of soluble factors that finally improve muscle strength. This possible mechanism could be mediated by an effect on satellite cells or another cell population that participates in muscle repair. One such population is a muscle-resident population of nonsatellite progenitor cells that are termed bipotent fibro/adipogenic progenitors and are located in the muscle interstitium and neighbor muscle-associated blood vessels. These cells could contribute to preventing muscle atrophy by secreting paracrine factors such as IL-6, IGF-1, and Wnt1 [52, 53].

Alternatively, MSC administration produces an anti-inflammatory response in several models and pathologies. Systemic inflammation can increase during obesity, resulting in increased proinflammatory cytokines such as IL-1*β* and TNF-*α* [54], which induce muscle wasting [55]. Therefore, it is possible that the presently obtained results are due to decreased TNF-*α* plasma levels, which, in turn, could decrease the atrophic effect on skeletal muscle.

## 5. Conclusions

The present study is the first to demonstrate that systemically administered MSCs could prevent the muscle wasting associated with HFD-induced obesity, by preventing the decreased MHC levels, increased UPP activity, increased myonuclear apoptosis, and oxidative stress.

## Supplementary Material

We describe the physical and biochemical parameters of experimental animals, anatomical features of skeletal muscle, and autophagy markers in mice Control, HFD or HFD treated with MSC.

## References

[1] Jagoe R. T., Lecker S. H., Gomes M., Goldberg A. L. (2002). Patterns of gene expression in atrophying skeletal muscles: response to food deprivation. *The FASEB Journal*.

[2] Bailey J. L. (2013). Insulin resistance and muscle metabolism in chronic kidney disease. *ISRN Endocrinology*.

[3] Bhatt B. A., Dube J. J., Dedousis N., Reider J. A., O'Doherty R. M. (2006). Diet-induced obesity and acute hyperlipidemia reduce I*κ*B*α* levels in rat skeletal muscle in a fiber-type dependent manner. *American Journal of Physiology—Regulatory Integrative and Comparative Physiology*.

[4] Sishi B., Loos B., Ellis B., Smith W., Du Toit E. F., Engelbrecht A.-M. (2011). Diet-induced obesity alters signalling pathways and induces atrophy and apoptosis in skeletal muscle in a prediabetic rat model. *Experimental Physiology*.

[5] Pette D., Staron R. S. (2000). Myosin isoforms, muscle fiber types, and transitions. *Microscopy Research and Technique*.

[6] Higashino K., Matsuura T., Suganuma K., Yukata K., Nishisho T., Yasui N. (2013). Early changes in muscle atrophy and muscle fiber type conversion after spinal cord transection and peripheral nerve transection in rats. *Journal of NeuroEngineering and Rehabilitation*.

[7] Cabello-Verrugio C., Morales M. G., Rivera J. C., Cabrera D., Simon F. (2015). Renin-angiotensin system: an old player with novel functions in skeletal muscle. *Medicinal Research Reviews*.

[8] Bodine S. C., Latres E., Baumhueter S. (2001). Identification of ubiquitin ligases required for skeletal muscle atrophy. *Science*.

[9] Clarke B. A., Drujan D., Willis M. S. (2007). The E3 ligase MuRF1 degrades myosin heavy chain protein in dexamethasone-treated skeletal muscle. *Cell Metabolism*.

[10] Eddins M. J., Marblestone J. G., Suresh Kumar K. G. (2011). Targeting the ubiquitin E3 ligase MuRF1 to inhibit muscle atrophy. *Cell Biochemistry and Biophysics*.

[11] Foletta V. C., White L. J., Larsen A. E., Leger B., Russell A. P. (2011). The role and regulation of MAFbx/atrogin-1 and MuRF1 in skeletal muscle atrophy. *Pflügers Archiv: European Journal of Physiology*.

[12] Arthur P. G., Grounds M. D., Shavlakadze T. (2008). Oxidative stress as a therapeutic target during muscle wasting: considering the complex interactions. *Current Opinion in Clinical Nutrition and Metabolic Care*.

[13] Kelley D. E., He J., Menshikova E. V., Ritov V. B. (2002). Dysfunction of mitochondria in human skeletal muscle in type 2 diabetes. *Diabetes*.

[14] Stump C. S., Henriksen E. J., Wei Y., Sowers J. R. (2006). The metabolic syndrome: role of skeletal muscle metabolism. *Annals of Medicine*.

[15] Meneses C., Morales M. G., Abrigo J., Simon F., Brandan E., Cabello-Verrugio C. (2015). The angiotensin-(1–7)/Mas axis reduces myonuclear apoptosis during recovery from angiotensin II-induced skeletal muscle atrophy in mice. *Pflugers Archiv*.

[16] Schrepfer S., Deuse T., Reichenspurner H., Fischbein M. P., Robbins R. C., Pelletier M. P. (2007). Stem cell transplantation: the lung barrier. *Transplantation Proceedings*.

[17] Phinney D. G., Prockop D. J. (2007). Concise review: mesenchymal stem/multipotent stromal cells: the state of transdifferentiation and modes of tissue repair—current views. *STEM CELLS*.

[18] Deans R. J., Moseley A. B. (2000). Mesenchymal stem cells: biology and potential clinical uses. *Experimental Hematology*.

[19] Hoogduijn M. J., Betjes M. G. H., Baan C. C. (2014). Mesenchymal stromal cells for organ transplantation: different sources and unique characteristics?. *Current Opinion in Organ Transplantation*.

[20] Rasmusson I. (2006). Immune modulation by mesenchymal stem cells. *Experimental Cell Research*.

[21] Caplan A. I., Dennis J. E. (2006). Mesenchymal stem cells as trophic mediators. *Journal of Cellular Biochemistry*.

[22] Deacon R. M. (2013). Measuring the strength of mice. *Journal of Visualized Experiments*.

[23] Morales M. G., Olguín H., Di Capua G., Brandan E., Simon F., Cabello-Verrugio C. (2016). Endotoxin-induced skeletal muscle wasting is prevented by angiotensin-(1–7) through a p38 MAPK-dependent mechanism. *Clinical Science*.

[24] Cabello-Verrugio C., Morales M. G., Cabrera D., Vio C. P., Brandan E. (2012). Angiotensin II receptor type 1 blockade decreases CTGF/CCN2-mediated damage and fibrosis in normal and dystrophic skeletal muscles. *Journal of Cellular and Molecular Medicine*.

[25] Morales M. G., Cabrera D., Céspedes C. (2013). Inhibition of the angiotensin-converting enzyme decreases skeletal muscle fibrosis in dystrophic mice by a diminution in the expression and activity of connective tissue growth factor (CTGF/CCN-2). *Cell and Tissue Research*.

[26] Morales M. G., Cabello-Verrugio C., Santander C., Cabrera D., Goldschmeding R., Brandan E. (2011). CTGF/CCN-2 over-expression can directly induce features of skeletal muscle dystrophy. *The Journal of Pathology*.

[27] Gregorevic P., Plant D. R., Leeding K. S., Bach L. A., Lynch G. S. (2002). Improved contractile function of the mdx dystrophic mouse diaphragm muscle after insulin-like growth factor-I administration. *The American Journal of Pathology*.

[28] Cisternas F., Morales M. G., Meneses C. (2015). Angiotensin-(1–7) decreases skeletal muscle atrophy induced by angiotensin II through a Mas receptor-dependent mechanism. *Clinical Science*.

[29] Meneses C., Morales M. G., Abrigo J., Simon F., Brandan E., Cabello-Verrugio C. (2015). The angiotensin-(1–7)/Mas axis reduces myonuclear apoptosis during recovery from angiotensin II-induced skeletal muscle atrophy in mice. *Pflugers Archiv—European Journal of Physiology*.

[30] Morales M. G., Abrigo J., Meneses C., Cisternas F., Simon F., Cabello-Verrugio C. (2014). Expression of the Mas receptor is upregulated in skeletal muscle wasting. *Histochemistry and Cell Biology*.

[31] Abrigo J., Rivera J. C., Simon F., Cabrera D., Cabello-Verrugio C. (2016). Transforming growth factor type beta (TGF-*β*) requires reactive oxygen species to induce skeletal muscle atrophy. *Cellular Signalling*.

[32] Le N. H., Kim C.-S., Park T. (2014). Quercetin protects against obesity-induced skeletal muscle inflammation and atrophy. *Mediators of Inflammation*.

[33] Lee S.-R., Khamoui A. V., Jo E. (2015). Effects of chronic high-fat feeding on skeletal muscle mass and function in middle-aged mice. *Aging Clinical and Experimental Research*.

[34] Shpilberg Y., Beaudry J. L., D'Souza A., Campbell J. E., Peckett A., Riddell M. C. (2012). A rodent model of rapid-onset diabetes induced by glucocorticoids and high-fat feeding. *Disease Models and Mechanisms*.

[35] Peluso I., Palmery M. (2016). The relationship between body weight and inflammation: lesson from anti-TNF-*α* antibody therapy. *Human Immunology*.

[36] Khodabandehloo H., Gorgani-Firuzjaee S., Panahi G., Meshkani R. (2016). Molecular and cellular mechanisms linking inflammation to insulin resistance and *β*-cell dysfunction. *Translational Research*.

[37] Plomgaard P., Nielsen A. R., Fischer C. P. (2007). Associations between insulin resistance and TNF-*α* in plasma, skeletal muscle and adipose tissue in humans with and without type 2 diabetes. *Diabetologia*.

[38] Li Y.-P., Chen Y., John J. (2005). TNF-*α* acts via p38 MAPK to stimulate expression of the ubiquitin ligase atrogin1/MAFbx in skeletal muscle. *FASEB Journal*.

[39] Carbó N., Busquets S., van Royen M., Alvarez B., López-Soriano F. J., Argilés J. M. (2002). TNF-*α* is involved in activating DNA fragmentation in skeletal muscle. *British Journal of Cancer*.

[40] Hao Y., Jackson J. R., Wang Y., Edens N., Pereira S. L., Alway S. E. (2011). *β*-hydroxy-*β*-methylbutyrate reduces myonuclear apoptosis during recovery from hind limb suspension-induced muscle fiber atrophy in aged rats. *American Journal of Physiology—Regulatory Integrative and Comparative Physiology*.

[41] Lessard S. J., Rivas D. A., So K. (2016). The AMPK-related kinase SNARK regulates muscle mass and myocyte survival. *The Journal of Clinical Investigation*.

[42] Fu X., Zhu M., Zhang S., Foretz M., Viollet B., Du M. (2016). Obesity impairs skeletal muscle regeneration through inhibition of AMPK. *Diabetes*.

[43] Messi M. L., Li T., Wang Z. M., Marsh A. P., Nicklas B., Delbono O. (2015). Resistance training enhances skeletal muscle innervation without modifying the number of satellite cells or their myofiber association in obese older adults. *Journals of Gerontology Series A: Biological Sciences and Medical Sciences*.

[44] Tedesco F. S., Dellavalle A., Diaz-Manera J., Messina G., Cossu G. (2010). Repairing skeletal muscle: regenerative potential of skeletal muscle stem cells. *The Journal of Clinical Investigation*.

[45] Bareja A., Billin A. N. (2013). Satellite cell therapy—from mice to men. *Skeletal Muscle*.

[46] Gao J., Dennis J. E., Muzic R. F., Lundberg M., Caplan A. I. (2001). The dynamic in vivo distribution of bone marrow-derived mesenchymal stem cells after infusion. *Cells Tissues Organs*.

[47] Lee R. H., Pulin A. A., Seo M. J. (2009). Intravenous hMSCs improve myocardial infarction in mice because cells embolized in lung are activated to secrete the anti-inflammatory protein TSG-6. *Cell Stem Cell*.

[48] Jose S., Hughbanks M. L., Binder B. Y. K., Ingavle G. C., Leach J. K. (2014). Enhanced trophic factor secretion by mesenchymal stem/stromal cells with Glycine-Histidine-Lysine (GHK)-modified alginate hydrogels. *Acta Biomaterialia*.

[49] Chen S., Murphy J., Toth R., Campbell D. G., Morrice N. A., Mackintosh C. (2008). Complementary regulation of TBC1D1 and AS160 by growth factors, insulin and AMPK activators. *Biochemical Journal*.

[50] Yamada S., Buffinger N., DiMario J., Strohman R. C. (1989). Fibroblast growth factor is stored in fiber extracellular matrix and plays a role in regulating muscle hypertrophy. *Medicine and Science in Sports and Exercise*.

[51] Rocheteau P., Chatre L., Briand D. (2015). Sepsis induces long-term metabolic and mitochondrial muscle stem cell dysfunction amenable by mesenchymal stem cell therapy. *Nature Communications*.

[52] Joe A. W. B., Yi L., Natarajan A. (2010). Muscle injury activates resident fibro/adipogenic progenitors that facilitate myogenesis. *Nature Cell Biology*.

[53] Boppart M. D., Lisio M. D., Zou K., Huntsman H. D. (2013). Defining a role for non-satellite stem cells in the regulation of muscle repair following exercise. *Frontiers in Physiology*.

[54] Debnath M., Agrawal S., Agrawal A., Dubey G. P. (2016). Metaflammatory responses during obesity: pathomechanism and treatment. *Obesity Research & Clinical Practice*.

[55] Cohen S., Nathan J. A., Goldberg A. L. (2015). Muscle wasting in disease: molecular mechanisms and promising therapies. *Nature Reviews Drug Discovery*.

