# Supplementary material for: High Fat Diet-Induced Skeletal Muscle Wasting Is Decreased by Mesenchymal Stem Cells Administration: Implications on Oxidative Stress, Ubiquitin Proteasome Pathway Activation, and Myonuclear Apoptosis
Source: Oxid Med Cell Longev. 2016 Aug 8;2016:9047821. doi: 10.1155/2016/9047821 (PMC4992759; doi:10.1155/2016/9047821)
Supplement: Supplementary file 1 — We describe the physical and biochemical parameters of experimental animals, anatomical features of skeletal muscle, and autophagy markers in mice Control, HFD or HFD treated with MSC. [file 9047821.f1.pdf]

Table S1

	30 weeks Control	30 weeks HFD	38 weeks HFD	
	untreated	untreated	Vehicle-treated	MSC-treated
Body weight (g)	27.2 ± 4.6	46.7 ± 3.5*	52 ± 2.8*	50.8 ± 3*
Plasma insulin (µg/l)	0.6 ± 0.1	4.3 ± 2.4*	8.2 ± 2.8*	6.9 ± 2.5*
Blood glucose (mg/dl)	121 ± 22	171 ± 13*	185 ± 18*	177 ± 12*
Serum cholesterol (mg/dl)	80 ± 15	222 ± 20*	230 ± 39*	225 ± 21*
Serum triglicerides (mg/dl)	74 ± 18	105 ± 24*	112 ± 22*	110 ± 18*

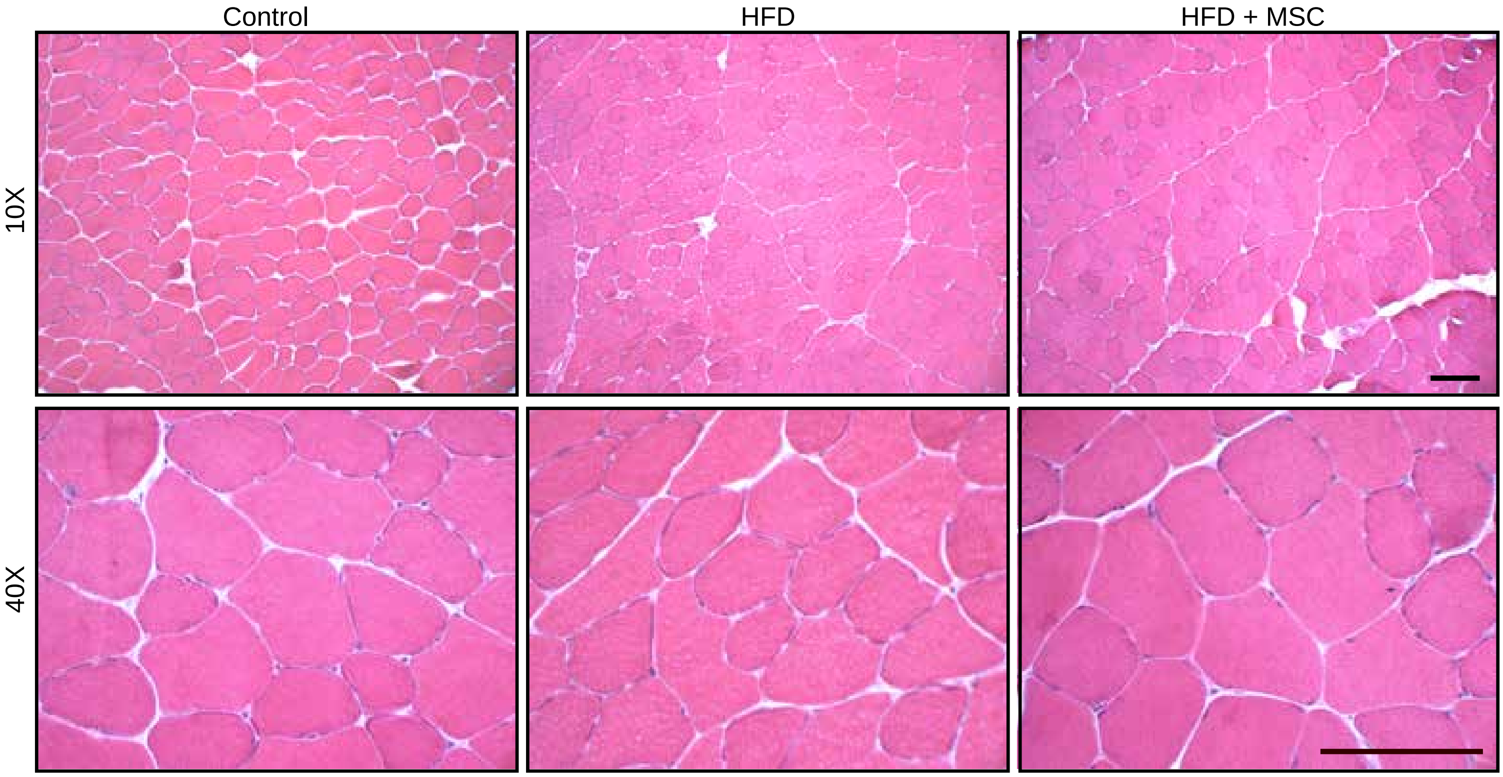

Fig. S1

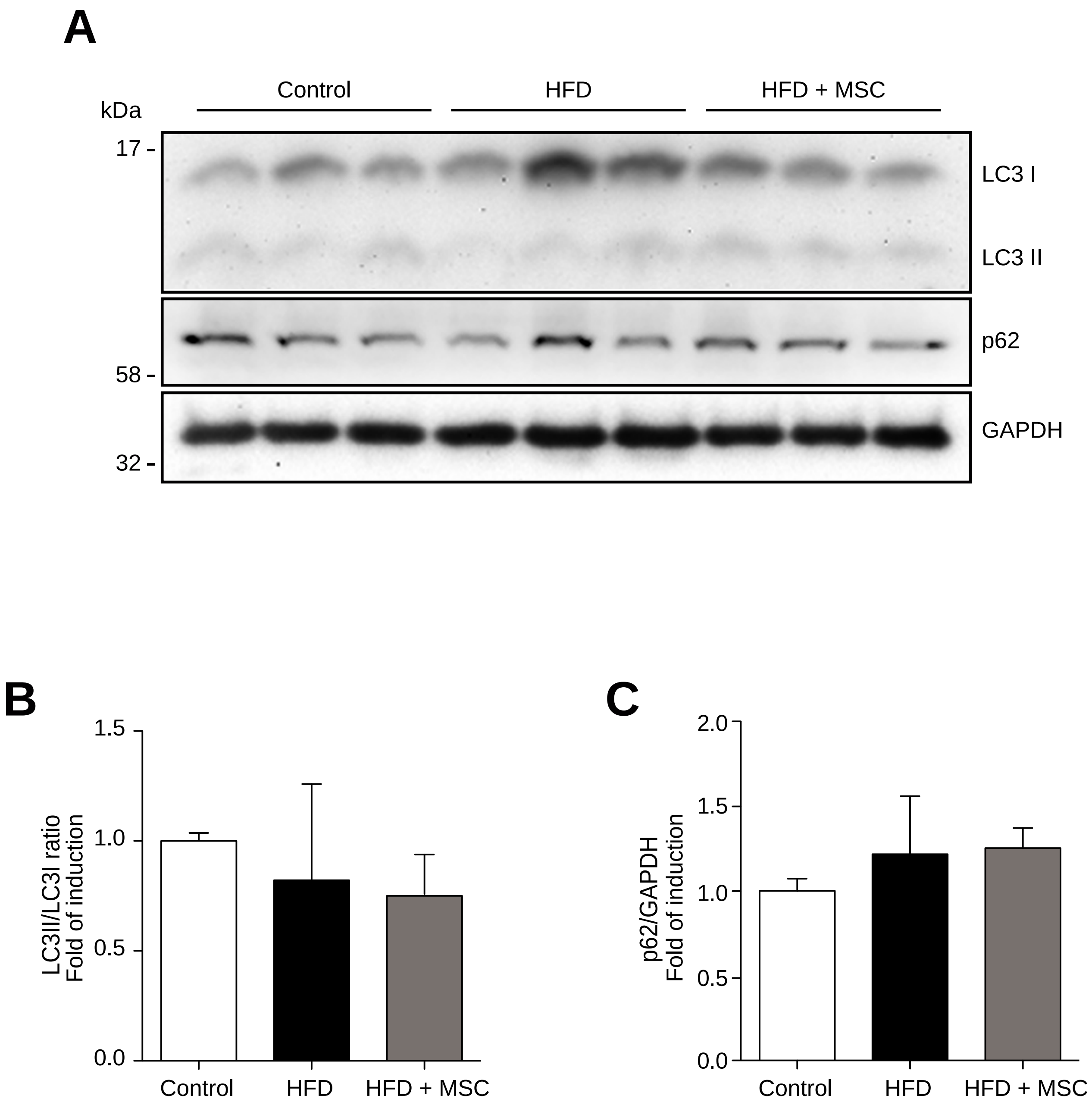
A
Control
HFD
HFD + MSC
kDa
17
58
32
LC3 I
LC3 II
p62
GAPDH
B
LC3II/LC3I ratio
Fold of induction
1.5
1.0
0.5
0.0
Control
HFD
HFD + MSC
C
p62/GAPDH
Fold of induction
2.0
1.5
1.0
0.5
0.0
Control
HFD
HFD + MSC

Fig. S2
